# Formaldehyde Crosses the Human Placenta and Affects Human Trophoblast Differentiation and Hormonal Functions

**DOI:** 10.1371/journal.pone.0133506

**Published:** 2015-07-17

**Authors:** Guillaume Pidoux, Pascale Gerbaud, Jean Guibourdenche, Patrice Thérond, Fatima Ferreira, Christelle Simasotchi, Danièle Evain-Brion, Sophie Gil

**Affiliations:** 1 INSERM, U1139, Paris, France; 2 Université Paris Descartes, Paris, France; 3 Université Paris Sud, EA4529, UFR de Pharmacie, Châtenay-Malabry Cedex, France; 4 APHP, Hôpital de Bicêtre, Service de biochimie, 94275 Le Kremlin Bicêtre, France; 5 PremUp, Paris, France; 6 Université Paris-Sud, Faculté de Pharmacie, Châtenay-Malabry Cedex, France; Shanghai Jiaotong University School of Medicine, CHINA

## Abstract

The chorionic villus of the human placenta is the source of specific endocrine functions and nutrient exchanges. These activities are ensured by the syncytiotrophobast (ST), which bathes in maternal blood. The ST arises and regenerates throughout pregnancy by fusion of underlying cytotrophoblasts (CT). Any anomaly of ST formation or regeneration can affect pregnancy outcome and fetal growth. Because of its direct interaction with maternal blood, the ST is sensitive to drugs, pollutants and xenohormones. *Ex vivo* assays of perfused cotyledon show that formaldehyde, a common pollutant present in furniture, paint and plastics, can accumulate in the human placenta and cross to the fetal compartment. By means of RT-qPCR, immunoblot and immunocytochemistry experiments, we demonstrate *in vitro* that formaldehyde exerts endocrine toxicity on human trophoblasts, including a decrease in the production of protein hormones of pregnancy. In addition, formaldehyde exposure triggered human trophoblast fusion by upregulating syncitin-1 receptor expression (ASC-type amino-acid transporter 2: ASCT2). Moreover, we show that formaldehyde-exposed trophoblasts present an altered redox status associated with oxidative stress, and an increase in ASCT2 expression intended to compensate for this stress. Finally, we demonstrate that the adverse effects of formaldehyde on trophoblast differentiation and fusion are reversed by N-acetyl-L-cysteine (Nac), an antioxidant.

## Introduction

The chorionic villus is the source of all placental functions during human pregnancy [1]. The villus is composed of a mesenchymal core that includes fetal capillaries and mesenchymal cells surrounded by mononuclear cytotrophoblasts covered by the syncytiotrophoblast, which bathes in maternal blood [2]. Feto-maternal exchanges of ions, nutrients and gases are ensured by the syncytial interface. The syncytiotrophoblast also has intense and specific endocrine functions, including the synthesis of protein (pGH: placental growth hormone, hPL: human placental lactogen, and hCG: human chorionic gonadotrophin) and steroid hormones (progesterone and estrogen). These hormones are necessary for the maintenance of pregnancy, maternal adaptation, fetal development and parturition [3,4].

Given its essential role and key location in the intervillous space, in direct contact with maternal blood, the syncytiotrophoblast is sensitive to drugs, xenobiotics and environmental pollutants absorbed by the mother, and this may have harmful consequences for syncytial function and regeneration and, thus, for fetal growth and development. Indeed throughout pregnancy, continuous regeneration of the syncytiotrophoblast is ensured by cytotrophoblast fusion to the existing syncytiotrophoblast. Purified cytotrophoblasts from human placenta cultured *in vitro* aggregate and fuse naturally to form non-proliferative multinucleated syncytiotrophoblasts that produce pregnancy-specific hormones [5].

Hypoxia and oxidative stress hinder human trophoblast fusion and differentiation [6,7], while numerous factors promote these processes, including cAMP, protein kinase-A (PKA) and epidermal growth factor (EGF) [8,9]. Syncytins (syncytin-1 and 2) are fusogenic retroviral envelope proteins highly expressed in the human placenta (see for review [10]). Syncytin-1 was the first protein shown to be involved in human trophoblast fusion [11]. To promote cell fusion, synctin-1 must interact with a specific receptor on the target cell membrane. Human sodium-dependent neutral amino acid transporter types 1 and 2 (ASCT1, ASCT2) are syncytin-1 receptors [12]. Human ASCT2 is more strongly expressed than hASCT1 in the placenta [13,14]. Syncytin-2, encoded by the Human Endogenous Retrovirus type FRD (HERV-FRD) envelope gene, is expressed in villous trophoblasts [15,16], and mediates trophoblast fusion via the major facilitator superfamily domain-containing receptor 2 (MFSD2) [17,18].

Formaldehyde (FA) is a common organic compound belonging to the aldehyde family, and is produced during metabolism of certain amino acids (serine, glycine) in humans and other animals. FA is essential for purine and thymidine biosynthesis [19]. It can be found at low concentrations in fruit, food and water, while common exogenous sources include vehicle exhaust, power plants, tobacco smoke, forest fires, decomposition of plant residues in soil, and photochemical oxidation of hydrocarbons. For more than half a century, FA has been widely used in resins (phenolics, urea and melamine), as adhesives and binders in particle boards, carpets and paints, as well as in the production of plastics, textile finishing, cosmetics, and insecticides [20,21]. The International Agency for Research on Cancer (IARC) and national agencies have classified FA as a human carcinogen (group 1) [20,21]. According to IARC, there is robust evidence of a causal link between FA exposure and the risk of nasopharyngeal cancer and leukemia [20,21]. Indeed, FA exposure and the oxidative stress it induces are cytotoxic and potentially carcinogenic [22].

Very few human studies have evaluated the effect of FA exposure on reproduction and development. Although controversial, some studies suggest reproductive and a developmental toxicity in both humans and mice. A meta-analysis of 18 human studies provided evidence that FA exposure is associated with miscarriage and possibly with other negative reproductive outcomes (low birth weight, premature birth and menstrual irregularities) [21,23,24]. For obvious ethical reasons, animal models are used to analyze the reproductive and developmental toxicity of formaldehyde. Male rats and mice exposed to FA present seminiferous tubule damage, decreased testosterone levels and decreased sperm counts, motility and viability. Female mice exposed to FA show hypoplasia of the uterus and ovaries as well as irregular estrus (for review see [21,23,24]). Moreover, female rats and mice exposed to FA present an increase in fetal abnormalities such as embryo degeneration, chromosome aberrations and aneuploidy. The size of the placenta and *corpus luteum* are decreased, as are fetal weight and size (for review see [21,23,24]). These observations support the reproductive and developmental toxicity of FA. Here we conducted *ex vivo* and *in vitro* studies to determine the effects of formaldehyde on trophoblast differentiation and functions. In human perfused placental cotyledon assays, we observed that placental exposure to formaldehyde led to rapid placental transfer and accumulation. Formaldehyde exposure of primary cultured trophoblasts promoted trophoblast cell fusion by increasing ASCT2 protein expression and also led to a decrease in trophoblast hormonal functions. Increased oxidative stress was observed in trophoblasts exposed to formaldehyde, an effect reversed by N-acetyl-L-cysteine (Nac).

## Materials and Methods

### Ethical statement

The study conformed to the Declaration of Helsinki. Placentas used for this study were obtained with the patients’ written informed consent, and the protocol was approved by our local ethics committee (CCPRB Paris Cochin n° 18–05). Placental tissues were obtained from women aged between 28 and 44 years with uncomplicated pregnancy undergoing normal Cesarean section (for *in vivo* studies) or vaginal delivery (for *ex vivo* studies) at the Cochin Port-Royal, Antony and Antoine Béclère hospital maternity units (Paris, France). Placentas were obtained from non-smoking women with a BMI between 18.1 and 28.5.

### Placental perfusion

Placentas from vaginal deliveries were perfused in an open double circuit using a validated method [25]. Maternal and fetal solutions were prepared with Earle medium containing 30 g/L of human serum albumin (LFB). A fetal artery and associated vein from a single placental cotyledon were cannulated and the fetal circulation was established at a flow rate of 6 mL/min. Two catheters were inserted into the intervillous space on the maternal side and the maternal circulation was established at a flow rate of 12 mL/min. The maternal and fetal compartments were adjusted to pH 7.4 ± 0.1 and pH 7.2 ± 0.1, respectively. Antipyrine (Sigma Aldrich), a freely diffusing marker used to control cotyledon permeability, was used at 20 mg/L, and formaldehyde (Sigma Aldrich) at 100 μM. [^3^H]-antipyrine (25 Ci/mmol, Hartmann Analytic) and [^14^C]-formaldehyde (50 mCi/mmol, Perkin-Elmer) were added to the maternal reservoir at 50 nCi/mL each. The perfusion assay was settle to 90 min and aliquots of media from the venous fetal circulation and the maternal reservoir were collected every 5 min. Following the perfusion assay, the cotyledon was washed in icecold PBS and sampled at 4°C to measure radioactivity. Samples were digested in 1 mL of Soluène (Perkin-Elmer) at 50°C and mixed with 10 mL of Ultima Gold counting fluid (Perkin-Elmer). Radioactivity was determined by liquid scintillation counting on a Beckman LS 6000 TA counter (Beckman). Fetal/maternal concentration ratios were calculated: they represent the fetal transfer rate (FTR) and are given as percentages. We also assessed the clearance index (FTR of formaldehyde / FTR of antipyrine) and the placental uptake ratio, calculated as follows: ([^14^C]-formaldehyde in cotyledon/[^14^C]-formaldehyde in the maternal compartment) x 100 and ([^3^H]-antipyrine in cotyledon/[^3^H]-antipyrine in the maternal compartment) x 100.

### Immunohistochemistry

Immunohistochemical analyses were performed on human placental biopsies as previously described [26]. Tissue samples were first fixed in 4% paraformaldehyde (PFA) for 4 h, followed by 1% PFA for 16 h, then embedded in 4% agarose. Blocks were cut into 120-μm thick sections using a Vibratome (Technical Products International). Sections were permeabilized for 30 min with 0.5% Triton X-100 and blocked for 2 h in 5% fatty acid-free bovine serum albumin (FFA BSA), 0.01% Triton X-100. A primary anti-cytokeratin7 (CK7) antibody (0.9 μg/ml; Dako) was prepared in PBS with 1% BSA and incubated with the sections overnight at 4°C, followed by a fluorochrome-conjugated secondary antibody (Alexa Fluor 555 (1:500; Life Technologies)). After washing, the samples were mounted in mounting medium with TO-PRO-3 for nuclear staining and photographed with a Leica TCS SP2 confocal microscope. Controls without primary antibody or with a non specific antibody of the same isotype were all negative.

### Trophoblast cell culture

Villous cytotrophoblasts were purified from placentas obtained after cesarean section, and cultured as previously described [27]. Villous tissue was dissected free of membranes, rinsed and minced in HBSS. The villous sample was then subjected to sequential enzymatic digestion in HBSS containing 0.25% trypsin (W/V), 5 IU/ml DNAse I, 25 mM HEPES, 4.2 mM MgSO4. Cell dissociation was monitored by light microscopy. The first two digests were discarded to eliminate residual syncytiotrophoblast fragments, and the cell suspensions resulting from the following four or five sequential digestions were pooled. The cells were then purified on a discontinuous Percoll gradient (5% to 70% in 5% steps). Cells that migrated to the middle layer (density 1.048–1.062 g/ml) were further purified by negative selection with a monoclonal anti-human leukocyte antigen A, B and C antibody as described elsewhere [28,29]. This antibody reacts with most cell types, including macrophages, fibroblasts and extravillous trophoblasts but not with villous cyto- or syncytiotrophoblasts. The isolated cells were plated at a final density of 140 000 per cm^2^ in DMEM containing 10% FCS and were incubated at 37°C with 5% CO_2_. Cells were plated in triplicate in 60-mm dishes and cultured for 3 days as previously described [7]. Cells were treated with formaldehyde (formaldehyde R.P. Normapur Prolabo) or hydrogen peroxide (H_2_O_2_, Gifrer) alone or with N-acetyl L cysteine (Nac, Sigma Aldrich) for 24, 48 or 72 h.

### Immunolocalization studies

Immunocytofluorescence staining was performed at 24, 48 and 72 h of culture. Cells were washed in PBS then fixed and permeabilized in methanol at -20°C for 8 min and blocked for 1 h in 1% FFA BSA. Primary monoclonal antibodies (2.5 μg each) to desmoplakin (DSP, 0.05 mg/ml, Abcam) and cytoDEATH M30 (cCK18, 0.5 μg/ml, Roche) were prepared in PBS with 1% FFA BSA and incubated with the cells overnight at 4°C. Secondary antibodies (Alexa Fluor 488 or 555) were diluted in PBS with 1% FFA BSA and incubated with the cells for 1 h at room temperature. Samples were mounted in mounted-medium with DAPI for nuclear staining and photographed with a BX60 epifluorescence microscope (Olympus) equipped with a 40x oil objective (Olympus 1.00), an ultrahigh-vacuum mercury lamp and a Hamamatsu camera (C4742-95). Pictures were analyzed with VisionStage Orca software (v 1.6).

### Cell viability

Cell viability was determined by trypan blue exclusion (Life Technologies) and caspase-cleaved CK18 immunostaining (cytoDEATH M30, 0.5 μg/ml, Roche) according to the manufacturers' protocols. Briefly, a 0.4% solution of trypan blue was added to the cell suspension at 0.04% final concentration. Blue-stained cells and total cells were counted immediately under a low-power microscope. Caspase-cleaved CK18 immunostaining was performed as previously described [30].

### Mononuclear cells and fusion assay

Syncytium formation was followed by monitoring the cellular distribution of desmoplakin and nuclei after fixation and immunostaining as previously described [8]. Desmoplakin staining at the boundaries of aggregated mononuclear cells gradually disappears during syncytium formation. Cell nuclei were counterstained with DAPI-containing mounting medium. From a random point in the middle of the coverslips, 1000 nuclei contained in desmoplakin-delimited mononuclear cytotrophoblasts and syncytia were counted. Three coverslips were examined for each experimental condition. Results are expressed as the number of nuclei per syncytium. The fusion index was determined as (N—S)/T, where N is the number of nuclei in the syncytia, S is the number of syncytia, and T is the total number of nuclei counted [31]. In the same way, the mononuclear cell ratio was determined as MC/T, where MC is the number of mononuclear cells and T is the total number of nuclei counted.

### Intracellular cAMP assay

Cells (3x10^6^) were plated in 60-mm dishes and cultured as described above. After 24 h, the cells were preincubated with 10 mM 3-isobutyl-1-methylxanthine (IBMX) for 1 h to prevent cAMP degradation and were then stimulated for 60 min with 100 μM formaldehyde. Cyclic AMP accumulation was quantified with the cAMP parameter assay kit (R&D systems KGE 002B) according to the manufacturer’s protocol. Cells were then frozen on dry ice and cAMP was extracted with ice-cold 65% ethanol. The extracts were dried and kept at -20°C until use. The cAMP concentration in the sample was determined by interpolation to a cAMP standard curve ranging from 0.42 to 8.57 pmol/mL.

### RNA extraction

Total RNA was extracted from primary trophoblast cells after 24 h of FA exposure by using the Trizol reagent (Life Technologies). The yield of extracted RNA was determined by measuring optical density at 260 nm. The purity and quality of extracted RNA were subsequently assessed by electrophoresis on 1% agarose gel with ethidium bromide staining. Only high-integrity RNA samples were used for PCR analysis.

### RT-polymerase chain reaction

Trophoblasts were exposed to formaldehyde, Nac or both for 24 h, and total RNA was then extracted. RT-PCR was performed with the RT^2^ profiler PCR array (SABioscience) or with Superscript III reverse transcriptase followed by amplification with Platinium Taq polymerase (Life Technologies), according to the manufacturers' protocols and as previously described [32]. The RT^2^ profiler PCR array contains an RT^2^ First Strand kit for RT assays and the RT^2^ SYBR Green/ROX qPCR Master mix. Briefly, 600 ng of total RNA was reverse-transcribed at 42°C for 15 minutes using the RT^2^ first strand kit (SABioscience). Reverse transcriptase was then inactivated by heating at 95°C for 5 minutes. Neosynthesized cDNA was added to 2x Syber PCR master mix and analyzed with a 384-well Human oxidativeStress and antioxidant PCR array (PAHS-065E-1—SABioscience). RT-PCR was performed using specific oligonucleotide primers based on the coding sequence of the hCG α (CGA) P1(+), 5’-TCCCACTCCACTAAGGTCCAA-3’; P1(-), 5’-CCCCATTACTGTGACCCTGTT-3’; hCG β (CGB) P2(+), 5’-GCTACTGCCCCACCATGACC-3’; P2(-), 5’-ATGGACTCGAAGCGCACATC -3’; hPL P3(+), 5’-GCATGACTCCCAGACCTCCTT-3’; P3(-), 5’-TGCGGAGCAGCTCTAGATTGG-3’; pGH P4(+), 5’-AGAACCCCCAGACCTCCCT-3’; P4(-), 5’-TGCGGAGCAGCTCTAGGTTAG-3’; syncytin-1 (HERV-W) P5(+), 5’-CGGACATCCAAAGTGATACATCCT-3’; P5(-), 5’-TGATGTATCCAAGACTCCACTCCA-3’; syncytin-2 (HERV-FRD) P6(+), 5’-GCCTGCAAATAGTCTTCTTT-3’; P6(-), 5’-ATAGGGGCTATTCCCATTAG-3’; ASCT2 P7(+), 5’-GGCTTGGTAGTGTTTGCCAT-3’; P7(-), 5’-GGGCAAAGAGTAAACCCACA-3’; MFSD2 P8(+), 5’-CTCCTGGCCATCATGCTCTC-3’; P8(-), 5’-GGCCACCAAGATGAGAAA-3’; PPIA P9(+), 5’-GTCAACCCCACCGTGTTCTT-3’; P9(-), 5’-CTGCTGTCTTTGGGACCTTGT-3’; GPx-3 P10(+), 5’-GTCTCCAACCACACTATCTAC-3’; P10(-), 5’-ACACACAATCACGCATACC-3’; GSR P11(+), 5’-AACATCCCAACTGTGGTCTTCAGC-3’, P11(-), 5’-TTGGTAACTGCGTGATACATCGGG-3’; OXSR1 P12(+), 5’-AGTTCATTGTTTGCCCTGCT-3’; P12(-), 5’-GCAAAGGGAGTCTACCACACA-3’. PCR was carried out using an ABI 7900 HT Fast Real instrument (Applied Biosystems). Universal cycling conditions were used (2 min at 50°C, 10 min at 95°C for the initial steps, then 15 s at 95°C, 1 min at 60°C for 40 cycles, with a dissociation step (95°C for 15 s, 60°C for 15 s, followed by a slow ramp to 95°C). Relative gene expression (ΔC_T_) was calculated by subtracting the signal threshold cycle (C_T_) of GAPDH from the C_T_ value of each studied gene. Subsequently, ΔΔC_T_ values were calculated by subtracting GAPDH ΔC_T_ (set as calibrator) from the ΔC_T_ of each individual gene and transformed by the 2^-ΔΔCT^ equation in order to obtain x-fold higher/lower studied gene expression. Changes (increase or decrease) smaller than 20% of control were not considered for analysis.

### Hormone assays

Total hCG and hPL concentrations were determined in supernatants after 24, 48 and 72 h of culture by using an ECLIA assay (Advia Centaur, Siemens) with a detection limit of 2 mU/ml for hCG and an ELISA kit (DiaSource KAPD 1283) with a detection limit of 0.043 mg/l for hPL.

### Protein preparation and immunoblot analysis

Total cell lysates were prepared in lysis buffer (Life Technologies; 50 mM Tris pH 7.4, 250 mM NaCl, 50 mM NaF, 5 mM EDTA, 1% Nonidet P40, 0.02% NaN3, 1 mM sodium orthovanadate) supplemented with a protease inhibitor cocktail (Merck) and a phosphatase inhibitor cocktail (Merck). Protein samples were resolved by SDS-PAGE and blotted onto nitrocellulose membranes. The filters were then blocked in 5% non-fat dry milk in Tris-buffered saline pH 7.4 (TBS) with 0.1% Tween 20 (TBS-T) for 45 min at room temperature and incubated overnight at 4°C with antibodies to actin (0.8 μg/ml, Sigma-Aldrich), GPx3 (1 μg/ml, Sigma-Aldrich), GSR (1 μg/ml, Sigma-Aldrich), catalase (0,5 μg/ml, Sigma-Aldrich), ASCT2/R syncytin 21–29 and syncytin-1 (1 μg/ml, gifts from Dr. Mallet), syncytin-2 (1 μg/ml, Abcam), MFSD2 (1 μg/ml, Santa Cruz), Superoxide dismutase-1 (SOD-1) and SOD-2 (1 μg/ml, StressGen), and hCG (1 μg/ml, Dako), oxidative stress-response 1 protein (OXSR1; 1 μg/ml, Santa Cruz). After washing in TBS-T and incubation with appropriate HRP-conjugated secondary antibodies, the blots were developed with Supersignal West Pico substrate (Thermo Scientific).

### Enzyme assays

Catalase, total SOD and total gluthatione peroxidase (GPx) activities were measured in control and treated cells as previously described [7,33].

### Statistical analysis

The StatView F-4.5 software package (Abacus Concepts, Inc.) was used for statistical analysis. Differences between groups were evaluated with Student’s unpaired t test or ANOVA, as appropriate. Post hoc analysis (Tukey) was used for individual comparisons and to obtain p values shown in the figure legends. The sample size and significance level is shown in the figure legends for each graph. All data are presented as means ± SEM unless otherwise stated. P < 0.05 was considered statistically significant.

## Results

### Formaldehyde crosses and accumulates in the human placenta

**Fig 1:**
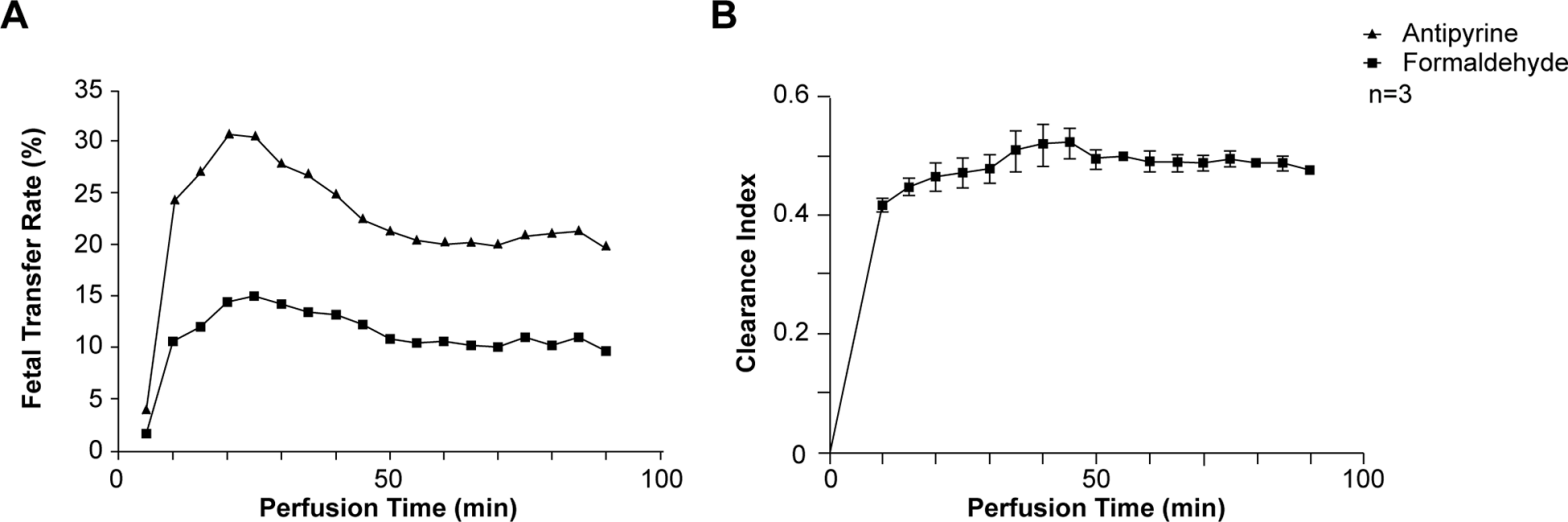
Characterization of transplacental formaldehyde transfer. (A) Fetal transfer rate of antipyrine (solid dark triangles) and formaldehyde (solid dark squares) and (B) clearance *versus* time, during 90 min of perfusion (mean ± SEM of 3 independent human perfused cotyledons).

To assess formaldehyde (FA) transfer from the maternal to the fetal compartment, human placental cotyledons were perfused with ^3^H-antipyrine and ^14^C-FA (Fig 1). Representative kinetics of the antipyrine and FA FTR are represented in Fig 1A. As expected, the antipyrine FTR increased rapidly in the first 20 min, then remained constant rate during the rest of the perfusion period. The total mean antipyrine FTR was 29.2 ± 9.8% (Table 1), demonstrating the overlap between the maternal and fetal circulation and validating the experiments. Interestingly, the FA FTR kinetics during the perfusion procedure had a profile similar to that of the antipyrine FTR (Fig 1A) and reached a plateau with an average value of 13.8 ± 4.7% (Table 1). The kinetics of the FA clearance index during the perfusion procedure is represented in Fig 1B. The mean clearance index increased during the first 10 min of perfusion and then plateaued for 90 min. The mean placental clearance index for FA was 0.47 ± 0.02, with a mean placental uptake ratio of 3 ± 1.4%; antipyrine did not accumulate in the placenta (Table 1). Together, these data suggest rapid transplacental FA transfer from the maternal to the fetal compartment, associated with trophoblast exposure to FA due to accumulation.

**Table 1:** Pharmacological characteristics of antipyrine and formaldehyde placental transfer during a 90-min period. The concentrations (%) of compounds added to the maternal compartment are indicated, along with the concentrations found in the fetal compartment after 90 min (FTR: fetal transfer rate). The clearance index is represented, as well as the percentage of compounds retained in the human cotyledon after 90 min of perfusion.

	Maternal compartment (%)	FTR (%)	Clearance Index	Cotyledon (%)
Antipyrine	100	29.2 ± 9.8	-	0
Formaldehyde	100	13.8 ± 4.7	0.47 ± 0.02	2.9 ± 1.4

### Formaldehyde exposure affects human trophoblast fusion

**Fig 2:**
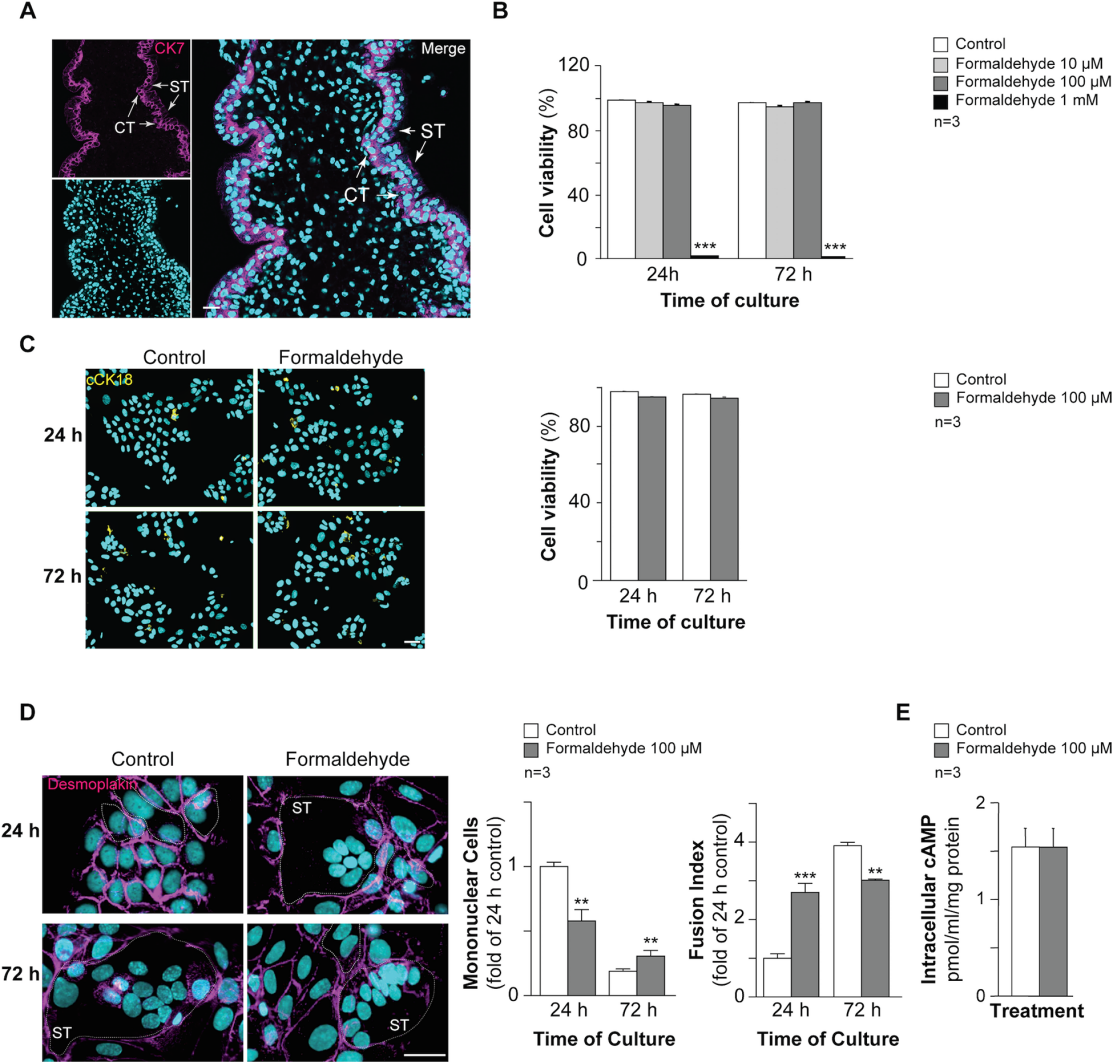
Effect of formaldehyde on human trophoblasts. (A) Immunohistofluorescence of cytokeratin 7 (CK7, magenta) in human placental biopsies; nuclei were counterstained with TOPRO-3 (cyan). Scale bar: 10 μm. (B) Histograms represent the viability (%) of untreated cells (control) and cells treated with formaldehyde (10, 50, 100 μM and 1 mM), as quantified with the Trypan blue exclusion assay. (C) Effect of formaldehyde exposure on human trophoblast apoptosis determined by immunocytofluorescence of cleaved cytokeratin 18 (cCK18, yellow) after 24 h and 72 h of culture; nuclei were counterstained with DAPI (cyan). Scale bar: 15 μm (left panel). Histograms represent viability (%) quantified by cCK18 immunostaining of untreated cells (control) and cells treated with 100 μM formaldehyde, after 24 h or 72 h of culture (right panel). (D) Immunocytofluorescence of desmoplakin (magenta) on human trophoblasts at 24 h and 72 h of untreated or formaldehyde-exposed (100 μM) culture; nuclei were counterstained with DAPI (cyan). Syncytia (ST) boundaries are indicated with dashed lines. Scale bar: 15 μm. (left panel). Effect of formaldehyde on cell fusion after 24 or 72 h of culture, represented as remaining mononuclear cells (middle left panel) and fusion index histograms (middle right panel). (E) Intracellular cAMP in control and formaldehyde-treated cells. Results are expressed as the mean ± SEM of 3 independent experiments (** p < 0.01, *** p < 0.001).

In order to establish the effect of FA exposure on trophoblast cell fusion, primary cytotrophoblasts (CT; positive for cytokeratin 7 (CK7, magenta, Fig 2A)) were purified from human placenta and cultured [32]. As previously reported, mononuclear cytotrophoblasts aggregated after 24 h of culture and then fused naturally to form multinucleated syncytia at 72 h, as shown by discontinuous desmoplakin immunostaining (Fig 2D) [26]. Primary cytotrophoblasts were cultured for 24 h or 72 h in the presence or absence of increasing concentrations of FA (from 10 μM to 1 mM) to determine the effect of the maximal non lethal FA concentration on trophoblast fusion and differentiation. Trophoblasts treated with 10 or 100 μM FA for 24 or 72 h showed no significant difference in viability compared to untreated cells in the Trypan blue exclusion assay (Fig 2B). Trophoblast exposure to 1 mM FA for 24 or 72 h resulted in more than 98% cell death (p < 0.001). Trophoblasts were treated for 24 or 48 h with 100 μM FA and cells positive for cleaved cytokeratin 18 (cCK18) were quantified by immunofluorescence as an early marker of apoptosis (Fig 2C). Histograms showed no significant changes in trophoblast viability after exposure to 100 μM FA for 24 and 72 h (Fig 2C). Thus, trophoblast exposure to 100 μM FA was not lethal and did not induce apoptosis. This concentration was therefore chosen to study the effect of FA on trophoblast cell fusion (Fig 2D). Trophoblasts treated with FA showed a significant decrease (above 1.5 fold; p < 0.001) in mononuclear cell numbers and an increase (approximately 2.5 fold) in syncytialization after 24 h of exposure compared to untreated cells. Conversely, exposure to FA for 72 h resulted in approximately 1.5-fold more mononuclear cells and approximately 1.3-fold less cell fusion (p < 0.001 versus untreated cells). As cAMP signaling is known to promote trophoblast fusion, intracellular cAMP was quantified in cells exposed to 100 μM FA (Fig 2E). No significant changes in intracellular cAMP production were noted. Together, these data suggest that trophoblast exposure to non lethal and non apoptotic FA concentrations affects cell fusion by promoting rapid syncytialization followed by steady-state cell fusion. This effect was not mediated by cAMP signaling.

### Formaldehyde affects human trophoblast hormonal functions

**Fig 3:**
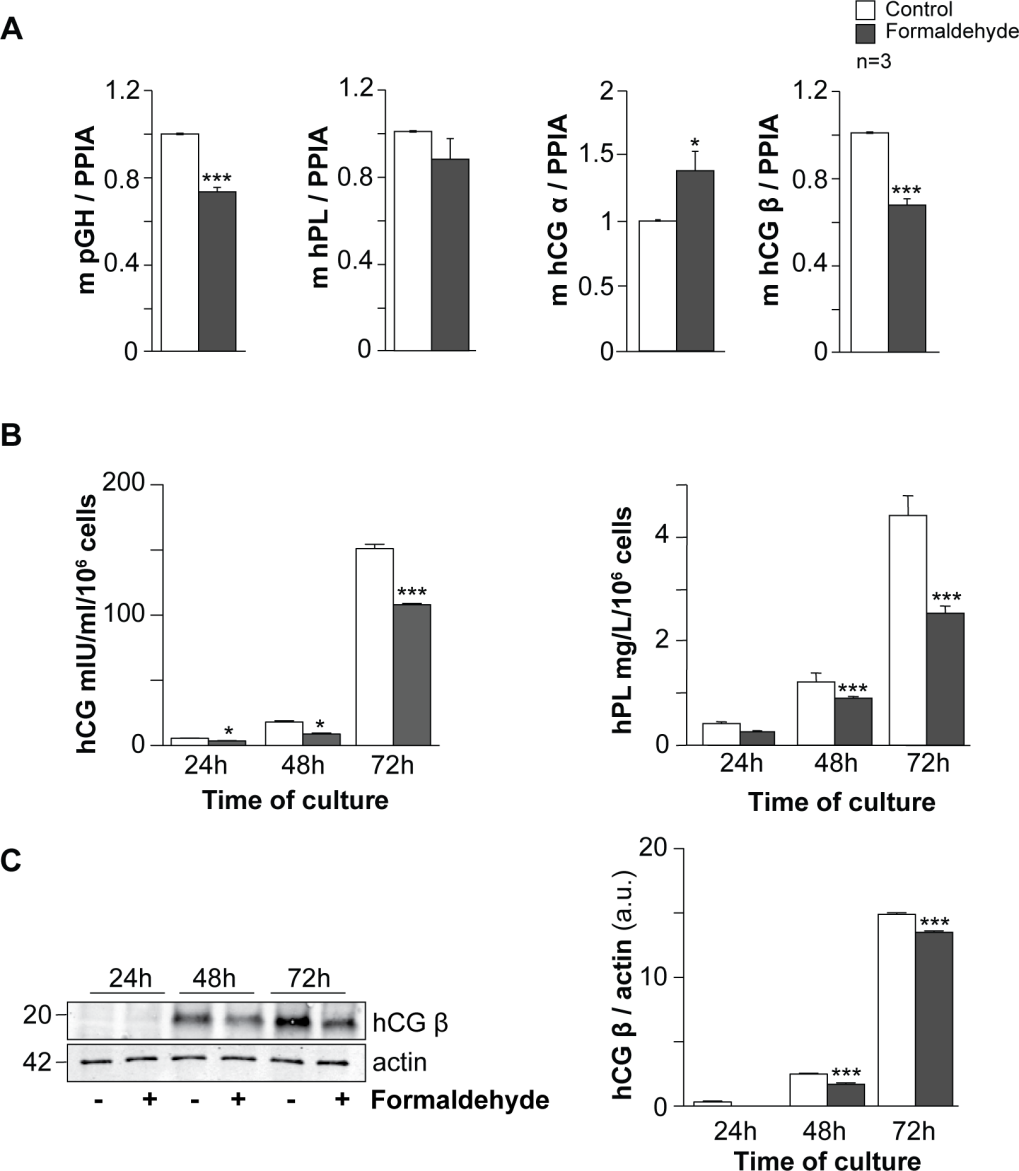
Effect of formaldehyde exposure on hormonal functions of human trophoblasts. (A) mRNA expression of peptide hormones of pregnancy (pGH, hPL, hCG α and β) in trophoblasts, with or without (control) formaldehyde exposure for 24 h. Data are normalized to cyclophilin A mRNA (PPIA). (B) Levels of total hCG (left histograms) and hPL (right histograms) secreted in the culture medium during trophoblast differentiation, with or without formaldehyde exposure. (C) Immunoblot analysis of hCG β and actin levels in untreated cells and cells exposed to formaldehyde during trophoblast differentiation (left panel). Levels of hCG β were assessed by densitometric scanning of immunoblots and normalized to actin levels in the same blots (right panel). Results are expressed as the mean ± SEM of 3 independent experiments (* p < 0.05, ** p < 0.01, *** p < 0.001).

Simultaneously with their syncytialization, differentiated villous trophoblasts develop hormonal functions, with increased synthesis and secretion of hormones of pregnancy such as pGH, hPL and hCG (Fig 3). To assess the effect of FA on trophoblast hormonal functions, mRNAs for pGH, hPL and hCG (α and β subunits) were quantified by RT-qPCR and normalized to PPIA (cyclophilin A) mRNA expression in trophoblasts exposed to 100 μM FA for 24 h (Fig 3A). Cells exposed to FA showed a significant decrease (approximately 30%) in pGH and hCG β mRNA expression (p < 0.001) compared to untreated cells. Exposed trophoblasts showed increased hCG α mRNA (up to 45%; p < 0.05) and no change in hPL mRNA expression compared to control cells (Fig 3A). *In vitro*, primary trophoblasts secrete hCG and hPL in large amounts (mg/ml), while pGH secretion remains discrete (ng/ml). Taking this into account, we focused on the levels of hCG and hPL secreted by trophoblasts in culture media. Interestingly, trophoblasts exposed to FA secreted significantly less total hCG and hPL during differentiation into a syncytiotrophoblast compared to untreated trophoblasts (p < 0.001) (Fig 3B). To determine whether the reduction in total hCG secretion resulted from intracellular hCG accumulation, immunoblots were performed on the same cultures as in Fig 2B (Fig 3C, left panel). At 48 h and 72 h of culture, FA-exposed trophoblasts showed reduced hCG β protein expression (approximately 30 and 10% normalized to actin levels at 48 h and 72 h respectively) compared to unexposed trophoblasts (p < 0.001) (Fig 3C right panel). These experiments clearly demonstrated that FA exposure affects specific trophoblast hormonal functions.

### Formaldehyde induces human trophoblast oxidative stress

**Fig 4:**
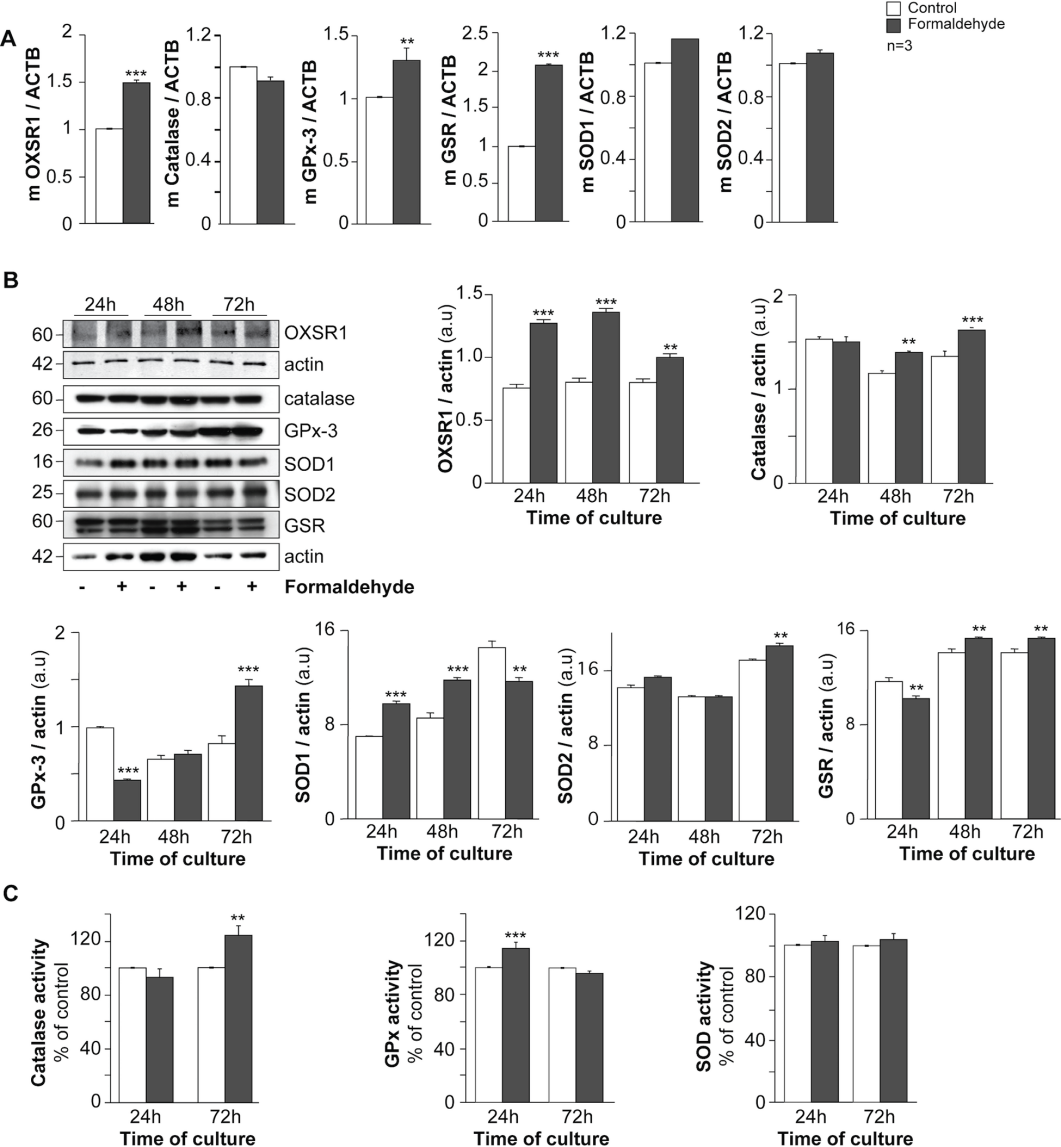
Effect of formaldehyde exposure on oxidative status in human trophoblasts. (A) The mRNA expression of OXSR1, catalase, GPx-3, GSR, SOD1 and SOD2 in trophoblasts with or without (control) formaldehyde exposure for 24 h. The mRNA data are expressed as the level of each mRNA marker normalized by actin beta mRNA expression (ACTB). (B) Immunoblot analysis of OXSR1, catalase, GPx-3, SOD1, SOD2, GSR and actin levels in cells with or without formaldehyde exposure during trophoblast differentiation (upper left panel). Levels of the proteins listed above were assessed by densitometric scanning of immunoblots and normalized to actin levels in the same blots (histograms; a.u. for arbitrary units). (C) Catalase, GPx and SOD activity in trophoblasts with or without formaldehyde exposure after 24 h or 72 h of culture. Results are expressed as the mean ± SEM of 3 independent experiments (** p < 0.01, *** p < 0.001).

Many studies have shown a correlation between FA exposure and oxidative stress [34]. We have previously demonstrated that oxidative status is critical for trophoblast fusion and hormonal functions [7,35]. In order to test whether FA exposure affects trophoblast functions by modifying intracellular oxidative status, trophoblasts were exposed to FA for 24 h and their oxidative status was studied (Fig 4). RT-qPCR analysis showed that FA-exposed trophoblasts exhibited higher OXSR1 (serine/threonine-protein kinase oxidative stress responsive 1), gluthatione reductase (GSR) and gluthatione peroxidase 3 (GPx-3) mRNA expression (50%, 100% and 40% respectively after normalization to actin beta mRNA: ACTB) compared to untreated trophoblasts (p < 0.001 and p < 0.01) (Fig 4A). In order to support these data, immunoblots were performed with FA-exposed and control trophoblast cultures (Fig 4B). FA-exposed trophoblasts showed a significant increase in OXSR1 after normalization to actin protein expression during the cell fusion process (p < 0.001 for 24 and 48 h and p < 0.01 for 72 h of culture). Likewise, FA exposure increased GSR (p < 0.01) and catalase protein expression after 48 h and 72 h (p < 0.01 and p < 0.001 respectively) after normalization to actin protein expression. Moreover, SOD1 protein expression increased after 24 h and 48 h (p < 0.001), and decreased at 72 h of culture (p < 0.01). SOD2 protein expression normalized to actin protein expression remained constant for the first 48 h and increased at 72 h of culture compared to unexposed trophoblasts (p < 0.01). Interestingly, GPx-3 protein expression normalized to actin protein expression decreased in 24 h FA-exposed trophoblasts (p < 0.001) and increased significantly at 72 h compared to control cells (p < 0.001; Fig 4B). In order to support these immunoblot results, oxidative enzyme activities were measured in FA-exposed trophoblasts and control cells (Fig 4C). Catalase activity increased significantly after 72 h of culture (above 20%, p < 0.01) in FA-exposed cells compared to control trophoblasts (Fig 4C, left histograms). Contrary to catalase activity, FA-exposed trophoblasts showed a significant increase in GPx activity at 24 h of culture (p < 0.001, approximately 20%) compared to control cells (Fig 4C, middle histograms). Finally, no differences in total SOD activity were noted between FA-exposed and unexposed trophoblasts (Fig 4C, right histograms). These data suggest that FA exposure affects the oxidative status of trophoblasts.

### Formaldehyde exposure promotes syncytialization through ASCT2

**Fig 5:**
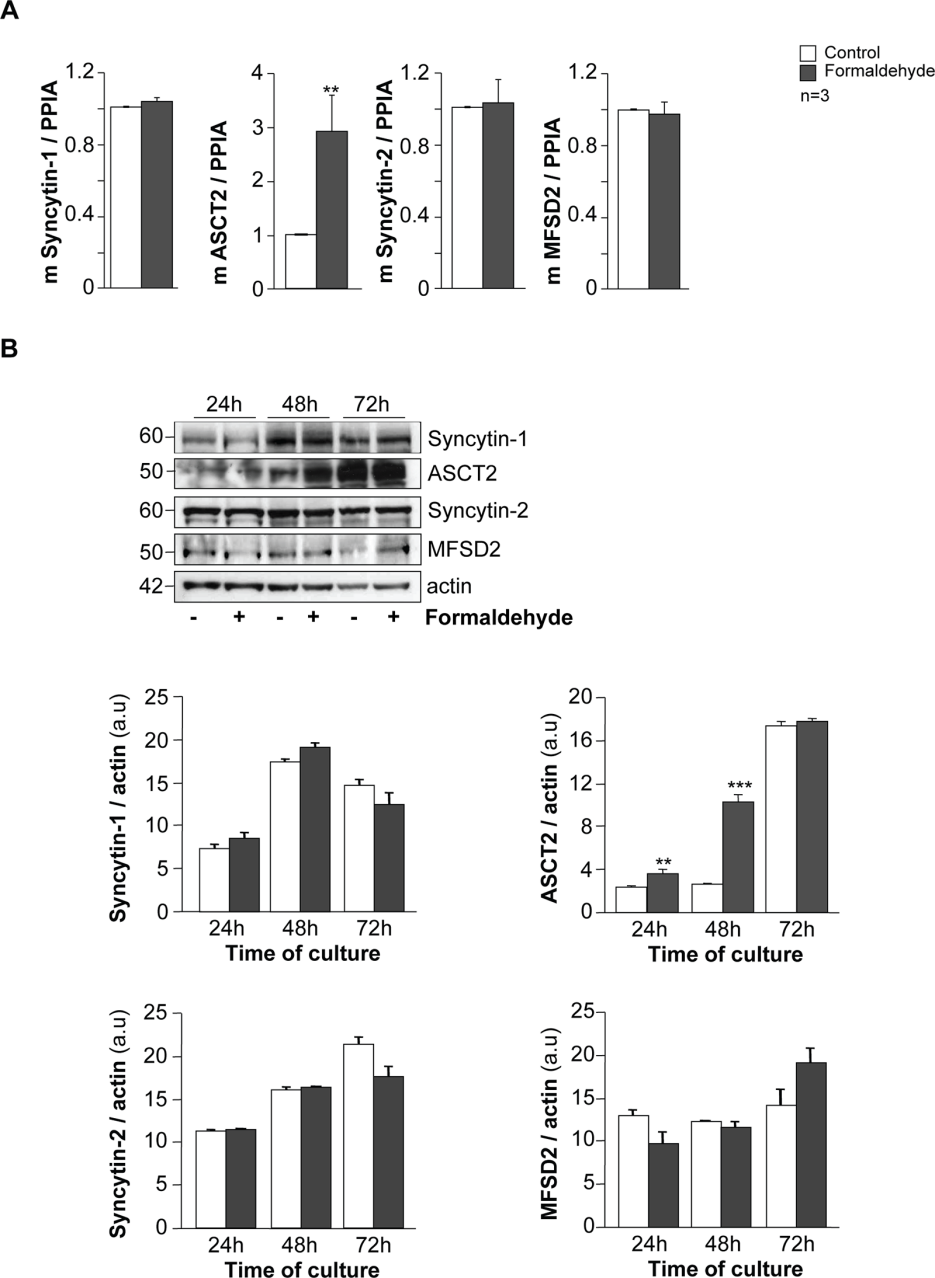
Effect of formaldehyde exposure on syncytins and syncytin receptors in human trophoblasts. (A) mRNA expression of syncytins and their receptors (syncytin-1, ASCT2, syncytin-2 and MFSD2) in trophoblasts with or without (control) formaldehyde exposure for 24 h. The mRNA data are expressed as the level of each mRNA marker normalized by cyclophilin A mRNA expression (PPIA). (B) Immunoblot analysis of syncytin-1, ASCT2, syncytin-2, MFSD2 and actin levels in cells with or without formaldehyde exposure during trophoblast differentiation (upper panel). Levels of the protein listed above were assessed by densitometric scanning of immunoblots and normalized to actin levels in the same blots (histograms; a.u. for arbitrary units). Results are expressed as the mean ± SEM of 3 independent experiments (** p < 0.01, *** p < 0.001).

To further assess the role of FA in the induction of trophoblast cell fusion, we studied the expression of syncytins and their receptors on 24 h FA-exposed and control trophoblasts (Fig 5). PPIA-normalized RT-qPCR performed on FA-exposed trophoblasts showed strong induction of ASCT2 mRNA expression (approximately 200%, p < 0.001) compared to unexposed trophoblasts, while syncytin-1, syncytin-2 and MFSD2 mRNA expression remained constant (Fig 5A). Immunoblotting of fusogenic proteins (syncytin-1 and syncytin-2) and receptor (MFSD2) (Fig 5B) normalized to actin showed no effect of FA during trophoblast fusion (Fig 5B). As observed for mRNA expression, an increase in ASCT2 protein expression was noted in FA-exposed trophoblasts at 24 h (p < 0.01, above 33%) and 48 h (p < 0.001, above 200%) compared to untreated cells (Fig 5B). Moreover, in normal conditions, syncytin-1 protein expression during the cell fusion process was maximal at 48 h of culture, while syncytin-2 protein expression increased gradually to a maximum at 72 h (Fig 5B). ASCT2 protein expression was maximal at 72 h (more than 5-fold higher than at 24 h). MFSD2 protein expression remained constant during the cell fusion process.

### The effects of formaldehyde on trophoblast differentiation are reversed by antioxidant treatment

**Fig 6:**
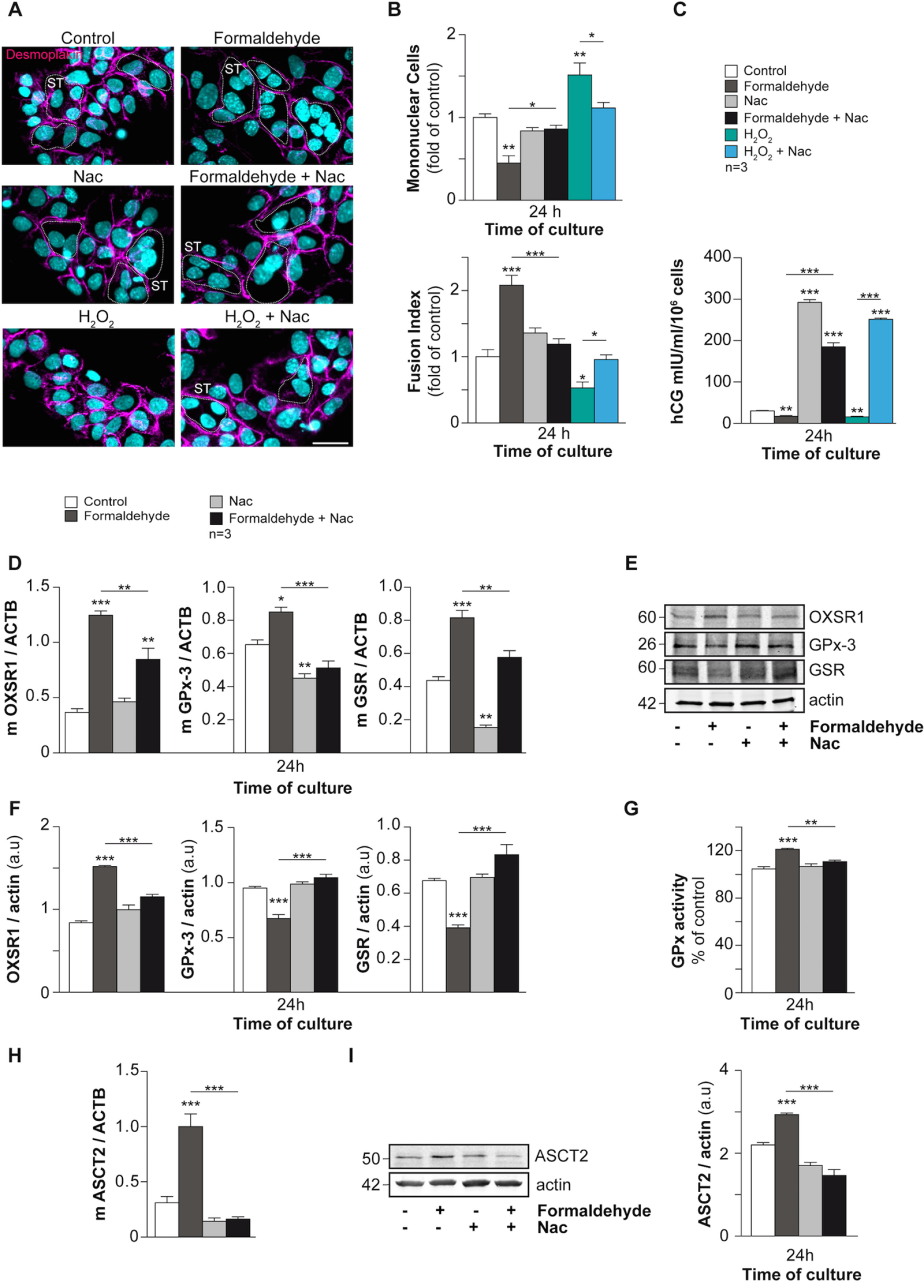
N-acetyl-L-cysteine (Nac) reverses the effects of formaldehyde on human trophoblast differentiation and fusion. (A) Immunocytofluorescence of desmoplakin (magenta) on human trophoblasts at 24 h of culture with or without formaldehyde, Nac, H_2_O_2_, or a combination of formaldehyde and Nac or H_2_O_2_ and Nac. Nuclei were counterstained with DAPI (cyan). Syncytia (ST) boundaries are indicated by dashed lines. Scale bar: 15 μm. (left panel). (B) Effect of formaldehyde, Nac and H_2_O_2_ alone or in combination on cell fusion after 24 h of culture, represented as remaining mononuclear cells (upper panel) and fusion index histograms (lower panel). (C) Levels of hCG secreted in the culture medium at 24 h in the same cultures. (D) The mRNA expression of OXSR1, GPx-3, GSR in trophoblasts exposed for 24 h to formaldehyde and/or Nac. The mRNA data are expressed as the level of each marker normalized to beta actin mRNA expression (ACTB). (E) Immunoblots of OXSR1, GPx-3, GSR and actin in the same conditions. (F) Analysis of previous immunoblots normalized to actin (a.u. = arbitrary units). (G) GPx activity measured in the same trophoblasts and conditions after 24 h of exposure. (H) The mRNA expression of ASCT2 normalized to beta actin mRNA expression (ACTB) in trophoblasts exposed for 24 h to formaldehyde and/or Nac. (I) Immunoblot analysis of ASCT2 and actin levels in the same conditions (left panel). Levels of the proteins listed above were assessed by densitometric scanning of immunoblots and normalized to actin levels in the same blots (histograms; a.u. for arbitrary units). Results are expressed as the mean ± SEM of 3 independent experiments (* p < 0.05, ** p < 0.01, *** p < 0.001).

To support the hypothesis that oxidative stress induced by FA triggers the increase in ASCT2 protein expression and cell fusion, trophoblasts were exposed to FA and/or N-acetyl-L-cysteine (Nac), an antioxidant. The effects of FA exposure or hydrogen peroxide (H_2_O_2_) alone or in combination with Nac were studied by cell fusion assay and hCG quantification. Antioxidant proteins, GPx activity and ASCT2 expression were also assessed in trophoblasts exposed to FA, alone or together with Nac (Fig 6). As shown by the disappearance of desmoplakin immunostaining (Fig 6A upper row) during syncytialization and fusion (Fig 6B), FA-exposed trophoblasts displayed a loss of mononuclear cells (Fig 6B upper histograms; above 2 fold, p < 0.01) and an increase in cell fusion (Fig 6B lower histograms; approximately 2 fold, p < 0.001) compared to control cells at 24 h culture (Fig 6A and 6B). These observations support our data shown in Fig 2. Interestingly, trophoblasts treated with FA concomitantly with Nac or with Nac alone displayed a return to normal cell fusion status at 24 h (p < 0.05), as shown by immunology (Fig 6A middle row) and fusion assays (Fig 6B). Moreover, trophoblasts cultured with H_2_O_2_ (50 μM) alone presented a significant decrease in cell fusion compared to controls at 24 h (approximately 0.5-fold for mononuclear cells and the fusion index, p < 0.01 and p < 0.05 respectively) (Fig 6A, lower row and 6B). Finally, coculture with H_2_O_2_ and Nac restored the cell fusion process (p < 0.05) (Fig 6A and 6B). As shown in Fig 3, FA-exposed cells showed a significant decrease in total hCG secretion (p < 0.01; Fig 6C), while cells treated with Nac in the presence or absence of FA showed an increase in total hCG secretion compared to control and FA-exposed cells after 24 h of culture (p < 0.001; Fig 6C). The addition of H_2_O_2_ led to a decrease in hCG secretion (p < 0, 01; Fig 6C), which was reversed by Nac (p < 0.001) (Fig 6C). OXSR1 and antioxidant (GPx-3 and GSR) mRNA and protein expression were assessed by RT-qPCR and immunoblots in control, FA- and/or Nac-exposed trophoblasts after 24 h of culture (Fig 6D and 6F). FA exposure promoted significant increases in OXSR1, GPx-3 and GSR mRNA (p < 0.001; p < 0.05; p < 0.001 respectively) (Fig 6D). Nac exposure decreased the expression of GPx-3 and GSR mRNA (p < 0.01 each), while OXSR1 mRNA remained unchanged (Fig 6D). Treatment with FA plus Nac restored GPx-3 and GSR mRNA expression (p < 0.001 and p < 0.01 respectively), while OXSR1 mRNA remained higher than control (p < 0.01) but was lower than after treatment with FA alone (p < 0.01) (Fig 6D). Trophoblasts exposed to FA for 24 h showed an increase in OXSR1 and a decrease in GPx-3 and GSR protein expression (p < 0.001 each) (Fig 6E and 6F). Trophoblasts incubated with Nac alone or in combination with FA exhibited a return of OXSR1, GPx-3 and GSR protein expression to basal level (Fig 6E and 6F). To support these observations, GPx activity was measured in the same trophoblasts exposed for 24 h to FA and/or Nac (Fig 6G). In the same way as Fig 4C, FA induced GPx activity by approximately 20% (p < 0.001) compared to control trophoblast cultures. Interestingly, trophoblasts cultured with Nac in combination with FA decrease GPx activity compared to FA-exposed trophoblasts (minus 20%; p < 0.01) and restored GPx activity at a similar level as in control (Fig 6G). Finally, ASCT2 protein expression was examined by RT-qPCR and immunoblot in control and FA- and/or Nac-exposed trophoblasts after 24 h of culture (Fig 6H and 6I). As shown in Fig 5, the level of ASCT2 mRNA and protein expression was significantly higher (p < 0.001 each) in FA-exposed trophoblasts compared to control cells (Fig 6H and 6I). Trophoblasts exposed to Nac alone or Nac plus FA showed a similar level as control of ASCT2 mRNA and protein expression (p < 0.001 each) (Fig 6H and 6I). These data suggest that antioxidant treatment with Nac can reverse the adverse effects of FA on trophoblast cell fusion and differentiation.

## Discussion

FA is known to promote nasal tumors and leukemia [20,21], but its reproductive and developmental toxicity are controversial [23,36–39]. Epidemiological studies have linked FA to spontaneous abortion, menstrual disorders, congenital malformations, low birth weight and infertility in both humans and mice [37–39]. However, other authors criticized the use of small groups or biased interpretation [36,40]. A recent histomorphological study concluded that FA induced toxic changes in mouse placental structure, leading to disruption of placental functions and decreased fetal weight [23]. Here, for the first time, effects of formaldehyde were observed in human placenta by using two complementary approaches, the *ex vivo* model of perfused cotydelon and primary cultures of human trophoblasts.

Although FA is an endogenous component of all biological systems, being produced in trace quantities, the toxicity of exogenous FA has become a serious public health issue. Heck et al. have reported a formaldehyde concentration in human blood controls of 2.61 μg/g [41]. This concentration is approximately 80–100 μM and was attributed to ubiquitous exogenous sources. In this context, it is noteworthy that the FA concentration used in the present study (*i*.*e*. 100 μM) was in the range of FA levels to which humans are commonly exposed. We found that this concentration of FA (100 μM) was the lowest non lethal concentration with cellular effects on human trophoblasts. This is in keeping with the concentrations reported not to induce cytotoxicity, genotoxicity, mutagenicity or apoptosis in other studies of various cell types [42,43].

The perfused human placental cotyledon model was used to study the kinetics of FA transfer across the placenta. This model is the only experimental technique which allows human placental transfer to be studied, an intact dual maternal-fetal circulation and placental tissue [44]. We observed rapid transplacental transfer of formaldehyde (FA) from the maternal to the fetal compartment. The main mechanism of placental transfer is passive diffusion. The transfer rate across the barrier is determined by the physicochemical properties of the molecule, such as lipid solubility, polarity, molecular weight, protein binding, and ionisation. The addition to the maternal circulation of a marker that undergoes only passive diffusion, such as antipyrine (ATP), can be used to measure tissue integrity/membrane permeability. Since ATP does not bind to proteins and does not accumulate in placental tissue, its transfer rate depends only on the fetal and maternal flows. Hence, a clearance index close to 1 demonstrates passive diffusion without placental accumulation [45]. The kinetics of FA transfer during the perfusion procedure was similar to that of antipyrine, with a clearance index of 0.47. FA can be metabolized and react with many biomolecules, such as proteins, nucleic acids and amino acids, and cause DNA-protein crosslinks and thereby accumulate in placental and fetal tissues. We observed accumulation of FA or its metabolites during a 90 min period. Our data are in agreement with those of Katakura et al., who reported that FA-injected pregnant mice displayed accumulation after 5 min in both the placenta and the fetuses [46–49]. This accumulation in human placenta could explain the adverse effects of FA exposure on human primary trophoblasts observed here, namely syncytiotrophoblast hormonal dysfunction and altered regeneration.

The endocrine activity of the human placenta is necessary to maintain pregnancy and to ensure fetal growth and development [3,4]. Placental environments containing toxics and pollutants have been reported to disrupt the endocrine activity of the human placenta [50,51]. Changes in syncytiotrophoblast mass, formation, regeneration or functioning can lead to abnormal endocrine production and provoke abnormal fetal development and miscarriage. The production and secretion of hCG is necessary at the beginning of pregnancy to induce progesterone synthesis by the ovarian *corpus luteum*, which leads to myometrium relaxation [52]. Human CG also acts in a paracrine and autocrine manner to trigger villous trophoblast differentiation and turnover throughout pregnancy [32]. Human PL and pGH are both involved in maternal adaptation to pregnancy and in the control of fetal growth [53,54]. Very few studies have focused on the effect of FA on the production and secretion of hormones in general, and none have specifically concerned pregnancy hormones. Rat and mice exposed to FA present reduced testosterone levels, suggesting an adverse effect of FA on hormone synthesis (for review see [24]). However, no cellular or biochemical studies have been performed to decipher the underlying mechanism. Here, we clearly demonstrated for the first time that FA exposure of human trophoblasts reduces pregnancy peptide hormone synthesis and secretion. A similar adverse effect was observed on hCG secretion after H_2_O_2_ treatment. Interestingly, we found that hormone secretion was restored by an antioxidant (Nac). We thus postulated that the oxidative stress induced by FA exposure, and particularly the H_2_O_2_ produced by FA metabolism, may trigger the adverse effects of FA on pregnancy hormone production. This is supported by a recent study in which H_2_O_2_ exposure affected hCG secretion by BeWo cells [55]. In keeping with previous studies, we found that *oxsr1* gene and protein expression in FA-exposed trophoblasts was increased, highlighting the induction of intracellular oxidative stress [34]. Oxidative stress or a redox imbalance due to FA exposure could be responsible for abnormal trophoblast functions [56]. For instance, trisomy 21 trophoblasts present SOD1 upregulation and unbalanced redox status, owing to the location of *sod1* on chromosome 21. This redox imbalance is associated with defective cell differentiation and hormone secretion [7,35,57]. It has been reported that exposure of pregnant women to FA is associated with spontaneous abortions and low birth weight, in keeping with our observation of syncytial dysfunction leading to abnormal trophoblast differentiation and regeneration and defective pregnancy hormone secretion.

FA also increased *GPx* and *GSR* gene expression, reflecting excessive consumption of glutathione (GSH) due to oxidative stress. In this context of FA exposure, both de novo synthesis and recycling of GSH are required to ensure the catalytic cycle of GPx, along with FA oxidation to formic acid. It is noteworthy that GPx-3 is a major peroxide scavenger in yeast, as deletion of this enzyme results in a dramatic increase in H_2_O_2_ sensitivity, contrary to deletion of the GPx-1 and GPx-2 isoforms [58]. We noticed a discrepancy between the GPx protein level and its activity in trophoblasts exposed to FA. This could be due to variations in the intracellular availability of GPx cofactors (selenium is the cofactor for GPx1-4 and 6). We have previously observed a similar discrepancy with SOD-1 protein expression and the intracellular SOD-1 cofactor (copper) concentration during trophoblast cell fusion [7,35,57]. Recent publications describe a protective effect of selenium against FA-induced oxidative stress [59,60]. However, more experiments are needed to quantify selenium during trophoblast differentiation and to confirm its protective effect against FA exposure of trophoblasts. It is interesting to note that catalase activity increased 72 h after FA exposure, compared to 24 h for GPx. This suggests that higher H_2_O_2_ concentrations are produced 72 h after FA exposure, an effect responsible for the diminution of cell fusion. Indeed, it is known that catalase metabolizes higher levels of hydrogen peroxide than GPx, without consuming additional GSH [61]. We found here that exogenous H_2_O_2_ decreased trophoblast fusion and differentiation. This is also supported by a study performed by Heazell and colleagues, who found that BeWo choriocarcinoma cells treated with H_2_O_2_ presented a decrease in cell fusion [55]. All these results suggest that excessive reactive oxygen species (ROS) are generated after FA exposure, inducing increased expression of antioxidant enzymes. This would be a compensatory mechanism for preventing cell damage, as observed in lung tissue after FA inhalation [34,62]. It is supported by the marked increase in ASCT2 gene and protein expression after 24 h. ASCT2 enables cell uptake of alanine, serine, cysteine and glutamine, which are involved in GSH synthesis. It has been reported in other cell types that increased ASCT2 expression facilitates uptake of glutamine, which is deaminated to glutamate by the mitochondrial glutaminase isoform 1 or 2 (GLS1 or GLS2). The newly synthesized glutamate is converted to GSH through activation of glutathione cysteine ligase (GCL), in order to reduce oxidative stress due to intracellular ROS and other free radicals [63–65]. Levovich et al. reported that an increase in intracellular FA in HL-60 and MCF-7 cells reduced the level of GSH by forming S-formylglutathione, resulting in increased ROS production [66]. The same effect was reported in isolated rat hepatocytes, and GSH depletion was related to FA metabolism and induction of lipid peroxidation [67]. Furthermore, FA is metabolized to formic acid by mitochondrial aldehyde dehydrogenase, an enzyme downregulated by high levels of female hormones such as estrogen and progesterone, suggesting that pregnant women may be more susceptible to FA exposure [68].

The deleterious effects of FA on trophoblast fusion, hCG secretion, redox status and ASCT2 mRNA and protein expression were reversed in the presence of Nac, an antioxidant. It is well known that Nac can be deacetylated into cysteine and further used to generate GSH, which, in association with GPx, is involved in peroxide detoxification. Nac may counteract the ASCT2-detoxifying pathway by producing GSH faster. Our data are in agreement with a previous study proposing Nac against cell damage induced by FA [69].

Interestingly, syncytin-1, syncytin-2 and MFSD2 expression remained unchanged in FA-exposed trophoblasts. The increased cell fusion observed in exposed cells was associated with the induction of ASCT2 mRNA and protein expression. In the human placenta, ASCT2 is the major receptor for syncytin-1, which promotes cell fusion. ASCT2 expression increased to counteract FA-induced ROS. Previous studies using siRNA and disrupting peptides have shown the crucial role of the syncytin-1/ASCT2 couple in the induction of cell fusion [11,70–72]. Pötgens et al. proposed several interesting models to explain the initiation of trophoblast cell fusion and how syncytin-1 and ASCT2 interconnect in mononuclear cells and syncytia [73]. In the most relevant model, the authors propose that syncytin-1 and ASCT2 are both expressed in fusion-competent cells. This steady state is characterized by the prevention of cell-cell fusion. This “receptor interference” checkpoint was previously described in retrovirus-cell fusion. Indeed, in neo-infected cells, the receptor for virus entry is blocked by the retroviral *env* protein. This phenomenon prevents subsequent infection by other retroviruses [74,75]. Syncytin-dependent cell-cell fusion appears to be triggered by upregulation of one or both partners (syncytin-1 and/or ASCT2) [73]. Our present findings support our previous studies showing that *in vitro* and in physiological conditions, syncytin-1 is expressed in cytotrophoblasts and syncytiotrophoblasts, peaking at 48 h of culture during the fusion process [76,77]. Interestingly, we found here that in physiological conditions ASCT2 was expressed in both cytotrophoblasts and syncytiotrophoblasts, with upregulation of expression in syncitia, in agreement with previous studies showing ASCT2 expression at the cytotrophoblasts and the syncytiotrophoblast basement membrane [76,78–80]. Our data suggest that in physiological conditions, upregulation of syncytin-1 at 48 h of culture alters the syncytin-1/ASCT2 balance and promotes trophoblast cell fusion, as previously reported [11]. The rapid increase in ASCT2 expression in FA-exposed trophoblasts led to an early syncytin-1/ASCT2 imbalance, which induces an early cell fusion process. However, more experiments are needed to characterize the molecular events that control the balance of syncytin-1/ASCT2 expression as a prerequisite for trophoblast fusion. The decrease in cell fusion observed in FA-exposed trophoblasts after 72 h of culture could be explained either by balanced expression of the two partners through the physiological increase in syncytin-1 protein expression, or to endocrine toxicity and the redox imbalance caused by FA.

In conclusion, this study provides new evidence supporting the toxicity of FA exposure during pregnancy. We propose a mechanism in which FA exposure has adverse effects on primary human trophoblasts. The few existing epidemiological studies have shown evidence of reproductive and developmental toxicity in pregnant women exposed to FA, with miscarriage and other adverse reproductive outcomes. In this study, we demonstrate that FA crosses the placental barrier from the maternal to the fetal circulation. This passage induces adverse effects on trophoblasts, including defective hormone production, induction of oxidative stress, and abnormal trophoblast differentiation, effects consistent with spontaneous abortion and adverse pregnancy outcomes observed in exposed populations. Our findings have major public health implications, particularly for pregnant women working or living in environments rich in FA. The use of antioxidants to reverse the adverse effects of FA raises interesting perspectives for treatment and/or prevention in pregnant women.
